# Diffusion-tensor Imaging and Tractography Application in Pre-operative Planning of Intra-axial Brain Lesions

**DOI:** 10.7759/cureus.1739

**Published:** 2017-10-03

**Authors:** Neetu Soni, Anant Mehrotra, Sanjay Behari, Sunil Kumar, Nishant Gupta

**Affiliations:** 1 Radiodiagnosis, SGPGIMS, Lucknow; 2 Sgpgims, SGPGIMS, Lucknow; 3 Radiology, Columbia University Medical Center

**Keywords:** keywords: brain tumor, diffusion tensor imaging, diffusion tensor tractography, corticospinal tract, motor function, arcuate fasciculus

## Abstract

Gliomas are the most common brain tumors that diffusely infiltrate the surrounding white matter (WM) tracts. Conventional MRI is commonly used for tumor localization and characterization. However, this does not give precise information about the WM infiltration surrounding the tumor. Diffusion-tensor imaging (DTI) is a non-invasive magnetic resonance (MR) technique that measures WM tissue integrity and tractography (fiber tracking) used to investigate the preferential directionality of diffusion. DTI allows visualization of WM tracts in the immediate vicinity of brain tumors that permit maximum tumor resection while also preserving the eloquent brain areas. The relation of tumors with the white matter tracts (deviation, infiltration, and disruption) has been one the most important initial applications of DTI. The fibers can be infiltrated in normal-appearing areas, and abnormal-appearing areas of the brain can show anatomically intact fibers. In the surgical planning of brain tumors, surgery is aided by knowing the proximity and relative position of the tumor to the adjacent WM tracts. The aim of the present study is to assess the role of DT tractography (DTT) in preoperative mapping of major WM tracts in relation to brain tumors.

## Introduction

In brain tumor patients, pre-operative surgical planning and assessment of the surrounding white matter (WM) tracts is a very important step to allow complete resection of tumor, while at the same time avoiding recurrence and loss of vital brain functions such as motor, sensory, auditory, language, and visual fields. Conventional MRI (cMRI) routinely demonstrates the lesion without any precise information about the involvement and integrity of the white matter tracts surrounding tumors [1]. Diffusion-tensor imaging with tractography (DTI) is currently the only available tool which displays the WM tracts disruption by fractional anisotropy (FA) changes and fibers reconstruction in patients with malignant brain tumors. Thus, it is very critical to accurately localize the WM tracts for preoperative surgical planning and making the decision of whether to operate or not [2].

DTI is derived from diffusion-weighted imaging (DWI), which is based on the random water diffusion in each voxel. However, the DTI adds directional information and provides three maps: quantitative apparent diffusion coefficient (ADC), the FA map, and the directionally encoded color-coded map (DEC).The ADC is a voxel-by-voxel measure of the magnitude of diffusion. FA measures preferential directionality of diffusion and expressed as a numerical value between 0 to 1. A high FA indicates a great degree of preferential directionality (anisotropy) such as in the highly organized WM tracts, and low FA indicates less preferential directionality such as in the gray matter (GM) and cerebrospinal fluid where FA is 0 (isotropy). The color-coded maps are produced from FA calculations, and by convention the blue color is used to highlight the tracts traveling in the inferior-superior direction, green in the anteroposterior direction, and red in the left-right direction. Fiber tracking can be performed with a multitude of different computational algorithms based on the preferential diffusion direction within voxels and allows 3 dimensions visualization of neural tracts [3-4]. The correlation between FA and tumor cell density is still controversial, as this effect causes a dilemma for neurosurgeons and might result in overestimating the destruction of neural fibers [5]. Many studies have assessed value of FA in predicting the destruction of fiber tracts in tumor-infiltrated WM areas by DTT [2, 6-7]. Three types of relationship were classified between tumor and tracts: a type with displacement only as type 1, a type featuring displacement with disruption as type 2, and a type with only disruption as type 3 [2].

Evaluating WM tracts injury by quantitative DTI is more helpful compared to morphological evaluation. The aim of the study is to show an important role of DTT in assessing tracts affected by the tumor, and to utilize this information in guiding surgeons to maximize the tumor resection, thus minimizing postoperative neurological deficit and chances of tumor recurrence.

**Figure 1:**
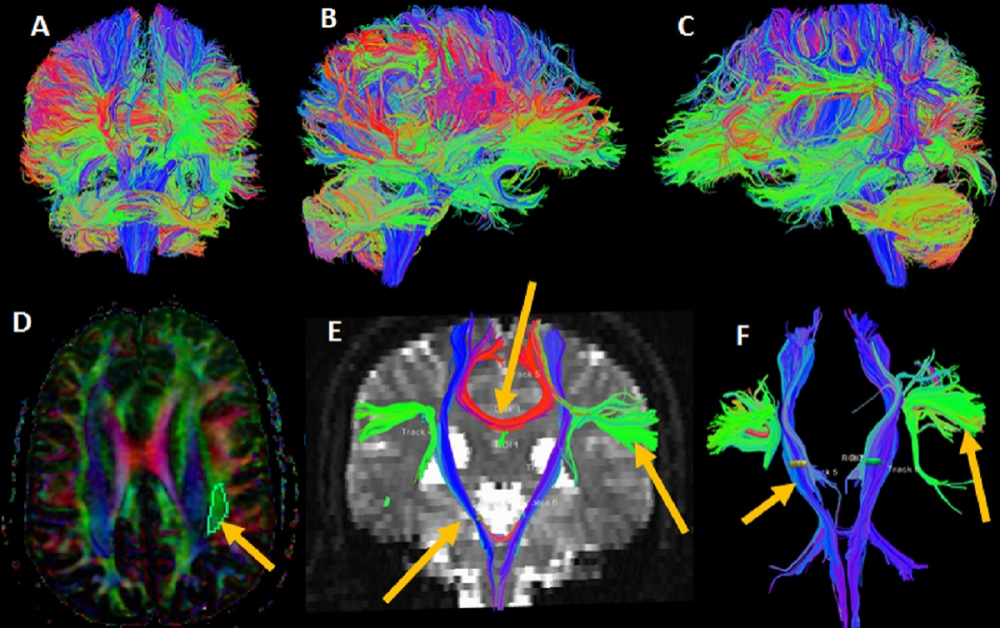
Whole-brain tractography Whole-brain tractography shows coronal (A), right sagittal (B), and left sagittal (C) views. Directionally-encoded color-coded map (D) shows green regions of interest (arrow) marked for left arcuate fasciculus. 3-D fiber tractography (E, F) of the blue-colored bilateral corticospinal tract (arrow) shows red-colored corpus callosum (arrow) and green-colored arcuate fasciculus fibers.

DTI data was processed using Diffusion Toolkit, a freely available software http://www.nmr.mgh.harvard.edu/~rpwang/dtk [8]. Whole-brain tractography and 3-dimensional reconstruction of selective WM tracts used a single region of interest (ROI) to assess the relationships between the tumor and eloquent areas. This is helpful in planning the safest surgical route and resection (Figure 1).

## Case presentation

Case 1

**Figure 2:**
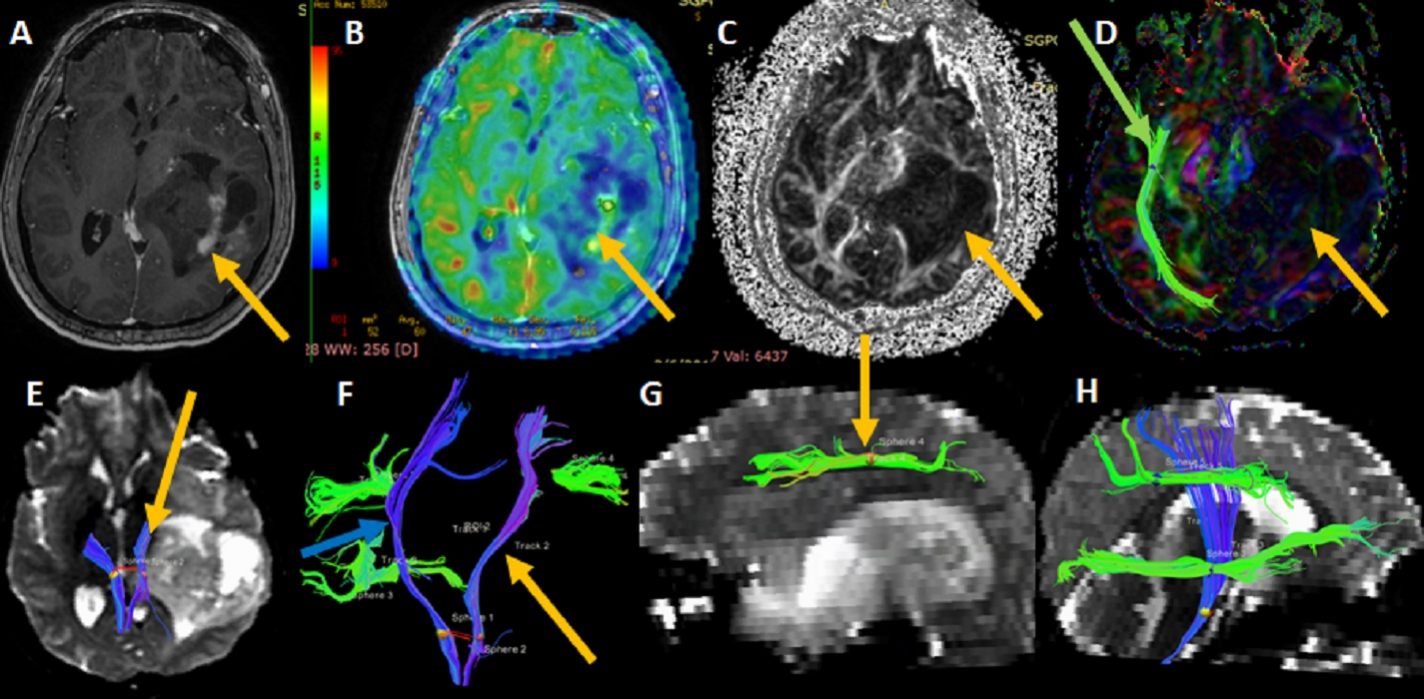
A 38-year-old male with left glioblastoma T1-weighted post-contrast image (A) shows the tumor in left temporal parietal region. Arterial spin labeling image (B) shows focal area of increased cerebral blood flow (arrow) within the enhancing area. Fractional anisotropy (FA) map (C) shows an intralesional decreased FA (arrow). Color-coded map (D) shows intralesional white matter tracts disruption of left inferior frontal-occipital fasciculus (IFO) tract (yellow arrow). The right IFO (green arrow) is intact and shown for comparison. 3-D fiber tractography (E, F) show blue-colored left CST (arrow) in close relation to lesion, and compressed and deviated anteromedially by the mass lesion. The right corticospinal tract (CST) is also displaced because of midline shift and mass effect. (G) The left green-colored arcuate fasciculus (arrow) is not affected by the lesion. (H) Normal intact left side fibers CST, IFO and AF (arcuate fasciculus) are seen.

A 38-year-old right-handed male presented with left glioblastoma, suffering from weakness in his right upper and lower extremities. MRI examination revealed an intra-axial tumor in the left temporal-parietal region. His language function was normal. The FA map showed decreased intralesional FA value, indicating the infiltration and destruction of WM tracts. The color-coded map showed complete disruption of the WM tracts of inferior fronto-occipital fasciculus (IFO) fibers. 3-D fiber tractography showed the intact left corticospinal tract (CST) in close relation to the lesion. The left CST was shown to be compressed and deviated medially by the mass lesion. The left arcuate fasciculus (AF) was seen away from the lesion, and unaffected by the same. Normal intact left-sided CST, IFO and AF tracts are shown for comparison. The tumor was resected maximally, avoiding the CST and AF, and was followed by chemoradiation (Figure 2).

Case 2

**Figure 3:**
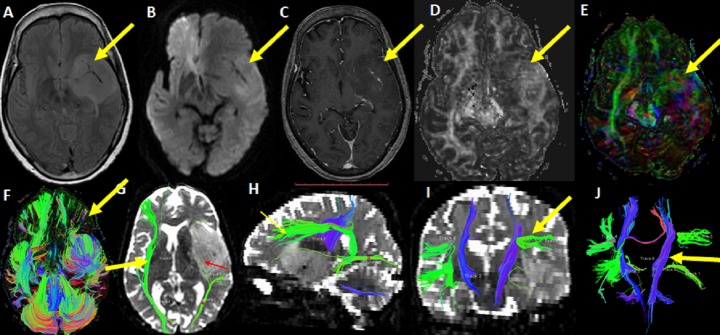
A 34-year-old female with left insular low grade glioma The FLAIR, (A) DWI (B), and post contrast T1-W (C) images show left insular lesion with no significant diffusion restriction as well as intralesional enhancement (arrows). Fractional anisotropy (FA) image (D) shows slightly- reduced FA. The colored map (E) shows distortion and destruction of white matter fibers in left frontal and temporal region (arrow). Axial whole brain tractography (F) shows destruction and distortion of the left frontal and insular fibers (arrow). 3-D tractography (G) shows destroyed left inferior frontal-occipital fasciculus (IFO) tract (red arrow) and intact right IFO (yellow arrow. 3-D tractography (H) shows anterior part of arcuate fasciculus (AF) in close relation to the anterior superior part of lesion (yellow arrow). (I) The leftcorticospinal tract (CST) is shown intact, away from lesion, yet medially deviated. 3-D tractography (J) of bilateral CST, AF, and IFO is shown.

A 34-year-old right-handed female developed an episode of partial epilepsy with transient language disorder. She recovered completely after few minutes of a seizure episode. MRI showed the left insular lesion characteristic for low-grade glioma. Pre-operative DTI was done to demonstrate the relationship of a lesion to eloquent areas to allow maximum tumor resection and to avoid tumor recurrence and damage to vital areas, such as language and motor function. Axial whole brain tractography showed destruction and distortion of the left frontal fibers and the left IFO tract. Anterior part of arcuate fasciculus (AF) was seen in close relation to the anterior-superior part of the lesion. The left CST was intact, yet medially deviated (Figure 3).

Case 3

**Figure 4:**
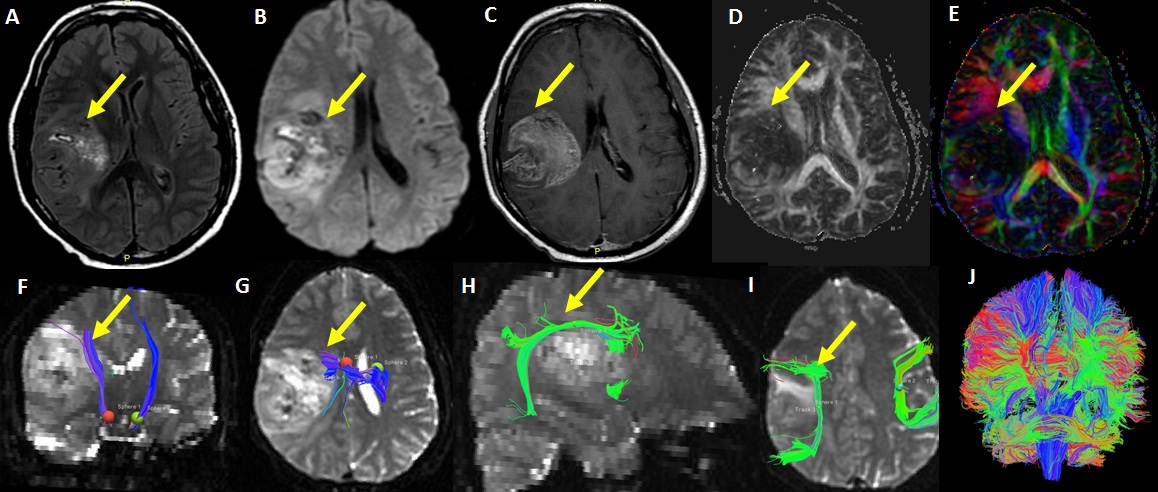
A 24-year-old male with right frontal-parietal glioblastoma FLAIR (A) DWI (B) Post contrast T1-W (C) images show a large enhancing tumor in the right frontal-parietal region. FA (D) and color-coded map (E) show decreased FA and destruction of white matter fibers. 3-D Tractography (F, G) was initiated from the brain stem (red and green sphere). The right corticospinal tract is shown passing through the anteromedial part of the lesion (arrow). The CST is also compressed and deviated anteromedially due to the lesion. 3D Tractography (H, I) of shows the arcuate fasciculus (arrow) compressed and deviated superiorly by the lesion and close to the tumor margin. Coronal whole brain tractography (J) of the same is shown.

A 24-year-old male presented with right frontal-parietal glioblastoma. The FA and color-coded maps showed decreased FA value and destruction of white matter fibers. 3D tractography was initiated from the brain stem for CST. The right CST passing through the periphery of the lesion was compressed and deviated anteromedially by the lesion. The AF was compressed and deviated superiorly by the lesion, and was close to the tumor margin (Figure 4).

## Discussion

High-grade gliomas are among the more aggressive tumors which infiltrate the surrounding WM and involve the eloquent brain areas. Visualization of the WM tracts by DTI is increasingly being used for neurosurgical planning. DTI allows pre-operative non-invasive 3D demonstration of the tracts which guide in surgical planning of the superficial and deep-seated brain lesions. Maximum resection of gliomas reduces the risk of tumor recurrence, while effective chemoradiation can result in loss of vital brain functions and poor quality of life. Pre-operative assessment of tumor extent is important for neurological outcomes, and has proved to be an independent major prognostic factor. The precise location of the WM tracts in relation to the lesion helps the neurosurgeon in preoperative planning by defining the surgical access point and identifying the extent of tumor resection while preserving vital motor, visual, or language brain functions. There are many imaging modalities such as conventional MRI, positron emission tomography, and functional MRI used to evaluate the brain tumors. MRI plays an important role in the diagnosis of different brain pathologies with the help of intravenous contrast administration to further characterize the lesion. The enhancement depends on the disruption of the blood-brain barrier which helps in targeting the biopsy in tumors. However, an involvement of the eloquent cortex and functional WM tracts in the brain is not demonstrated by the conventional MRI. Functional MRI (fMRI) demonstrates important motor and speech cortical areas by using special paradigms but does not give accurate WM tracts involvement [1-2]. DTI provides in-vivo tissue microstructure information by measuring water diffusivity for a given voxel in terms of FA and MD. The first neural tract images were produced in 1991 by Aaron Filler & colleagues, and the clinical utility of DT tractography (DTT) was identified in 2002 for patients with brain tumors [9]. Damage to motor CST and language AF tracts during surgery correlate with postoperative motor and language deficit. Yu CS, et al. demonstrated the relationship of tracts and tumors followed by post-operative DTI to investigate the surgical outcomes in 16 brain tumor patients. The authors found the extent of tumor resection and postoperative improvement of locomotive function of DTT group to be significantly higher than those of control group [2]. Min ZG, et al.* *reported that decreased FA and closeness of CST to malignant brain tumors are optimal factors in predicting the CST injury caused by brain tumors [10]. DTI can be acquired in intra-operative rooms and in some cases, preoperative unidentifiable CST were trackable on intraoperative scans due to change in local mass effects by the tumor on adjacent tracts [1]. DTI-based surgical planning in selective patients can present an alternate to awake surgery.

## Conclusions

Diffusion tensor imaging is a valuable tool in modern neurosurgery, as it can identify multiple WM pathways involved by intra-axial tumors. Thus this technique plays an important role in neurosurgical planning and in predicting the extent of safe resection. However, there are high variabilities in the sophistication of this modality, which limits interpretation of the results, thus warranting more studies to optimize and simplify fiber tracking. Progressive improvement in scanning as well as software-based methods of fiber tracking can overcome these limitations and help in neurosurgical planning.
